# Antibiotic Antibiogram in Patients With Complicated Urinary Tract Infections in Nephrology Unit of South Waziristan

**DOI:** 10.7759/cureus.29803

**Published:** 2022-10-01

**Authors:** Behzad Kaleem Baloch, Kifayat Ali, Nayab Memon, Shahzad Hassan, Mohammad Sohail Jan, Jibran Bin Aziz, Saba Kaleem

**Affiliations:** 1 Nephrology, Sheikha Fatima Bint-e-Mubarak Model Hospital, South Waziristan, PAK; 2 Internal Medicine, Sheikha Fatima Bint-e-Mubarak Model Hospital, South Waziristan, PAK; 3 Obstetrics and Gynaecology, Sheikha Fatima Bint-e-Mubarak Model Hospital, South Waziristan, PAK; 4 Microbiology, Institute of Basic Medical Sciences, Peshawar, PAK; 5 Public Health, Trans Continental Pharma Health, Peshawar, PAK; 6 Nephrology, University Hospital Limerick, Limerick, IRL; 7 Pathology, Combined Military Hospital Multan, Multan, PAK

**Keywords:** staphylococcus aureus, recurrent infection, antibiotic resistance, escherichia coli, complicated urinary tract infection

## Abstract

Objectives

To evaluate the antibiotic antibiogram in patients with complicated urinary tract infections (cUTIs) presenting to a Nephrology unit of South Waziristan.

Methods

A cross-sectional study was conducted at the Department of Nephrology, Sholam, South Waziristan. The study included all patients who presented with cUTIs and the symptoms included urinary urgency, hematuria, dysuria, suprapubic discomfort, and increased frequency. Those patients with clinical manifestations but are on antibiotics within the past five days were excluded.

Results

A total of 158 patients were included in the study with 113 (71.5%) females and 45 (28.5%) males. A total of 95 (60%) cases had gram-negative microbes, 47 (30%) had gram-positive cocci, and 16 (10%) had candida infection. In our study, the highly prevalent uropathogenic gram-positive bacteria showed the highest sensitivity to Linezolid, Rifampicin, and Vancomycin. Methicillin-resistant staph aureus was detected in 25% of isolates. All isolates of candida were sensitive to fluconazole. Gram-negative bacteria were highly resistant to ceftazidime, cefepime, ceftriaxone, and ciprofloxacin.

Conclusion

The development of bacterial resistance against multiple antibiotics is a global crisis that restricts the drug of choice for the treatment of cUTIs. In our study, we showed that overall, E.coli (gram negative) and S. Aureus (gram-positive) showed variable resistance to many antibiotics including ceftazidime, cefepime, piperacillin-tazobactam, ceftriaxone, and clindamycin.

## Introduction

Complicated urinary tract infections (cUTIs), the most frequent cause of hospital admissions, are also one of the major causes of morbidity and high medical costs [1]. Treatment of cUTIs can be challenging for doctors due to the rising incidence of antibiotic resistance and the lack of well-designed clinical studies [2].

People with cUTIs tend to experience recurring infections and need many rounds of antibiotic therapy. Additionally, patients may get an infection during their hospital stay through instrumentation, and organisms encountered in such an environment are more likely to be resistant to treatment than those contracted in the general population [3,4]. Globally, uropathogens are showing higher rates of resistance, according to the SENTRY Antimicrobial Surveillance program. Particularly, the rising prevalence of AmpC-lactamases and extended-spectrum-lactamase (ESBL)-producing microbes raises concerns about multidrug resistance [4].

The prevalence of antimicrobial resistance is more common when complicating factors are present and this often makes the response to therapy unsatisfactory, even with the drugs active against the causative pathogen, therefore it is crucial to distinguish between complicated and uncomplicated forms of urinary tract infection (UTI) [5]. Furthermore, serious complications like urosepsis, renal scarring, or even end-stage renal disease can occur [6,7].

In Pakistan and other regional countries, antibiotic resistance is becoming a serious problem [8]. Clinical isolates of E. coli have shown high rates of resistance to amoxicillin with clavulanic acid (20.6% - 27.9%), ciprofloxacin (64.7% - 74%), and piperacillin (71.1% - 80.1%). However, it is important to acknowledge antibiotic resistance patterns throughout Asia because the distribution of urinary pathogens and their susceptibility to antibiotics show regional heterogeneity [4,9]. In the present study, the antibiotic antigram in patients with cUTIs has been evaluated.

## Materials and methods

A cross-sectional study was conducted at the Department of Nephrology, Category D Hospital Sholam, South Waziristan, between March 2021 to June 2022. The study was started after ethical approval was obtained from the institutional review board (IRB) & ethical committee (EC) with the approval reference no of IRB#638-903-2021. The letter mentioned that the protocol was in accordance with the International Committee on Harmonization (ICH) and good clinical practice (GCP) guidelines and that any changes in the protocol should be notified to the committee for prior approval. A non-probability convenience sampling technique was implied to recruit participants.

The study included all patients who presented with complicated urinary tract infections (cUTIs) and the symptoms included urinary urgency, hematuria, dysuria, suprapubic discomfort, and increased frequency. An individual was diagnosed with a case of cUTI when he or she has a persistent urinary tract infection (UTI), treatment failure, and recurrences [10]. 

Those patients with clinical manifestations but are on antibiotics within the past five days were excluded. The sample size was determined using select statistics software by keeping a confidence level of 95% and a margin of error of 5%. The prevalence of UTIs in Pakistan was 11.6% as reported by Anwar Ullah et al., [11], which gave a sample size of 158.

Informed verbal and written consent was requested from all patients prior to the collection of the data. Each consenting patient's clean catch midstream urine was collected and placed in a 20 mL calibrated sterile screw-capped universal container that was given to the patients. Within 30 minutes of collection, each specimen was properly tagged and brought to the lab. All patients received thorough instruction from the researchers on how to collect clean catch midstream urine prior to sample collection. The sample was screened by gram staining. The semi-quantitative methodology was used to perform urine culture [12]. On blood agar and Mac Conkey's agar, urine (0.001ml) was cultivated with the use of a calibrated bacteriological loop. Gram reactions, activity, and biochemical traits were used to identify pathogenic microorganisms in accordance with standard microbiological procedures [13]. Only urine cultures were used to treat patients with cUTIs while blood cultures were not done. All the patients who fulfilled the criteria of cUTIs were screened for different anatomical abnormalities and risk factors causing resistant and recurrent infections. Different imaging modalities like ultrasound kidney and urinary bladder (KUB), urethrograms, voiding cystourethrograms, and prostatic ultrasound with post-void residual urinary volume were employed to diagnose underlying diseases like urethral and ureteral strictures, benign prostatic hypertrophy (BPH), reflux nephropathy, neurogenic bladder, etc, but results were not included in the study as it was beyond the scope of the study.

The SPSS (IBM Corp. Released 2015. IBM SPSS Statistics for Windows, Version 23.0. Armonk, NY: IBM Corp) was used to analyze the data. The main result was a pattern of antimicrobial sensitivity determined by the culture collected from each patient. The frequency and proportions of the several isolated microorganism types were presented.

## Results

**Figure 1:**
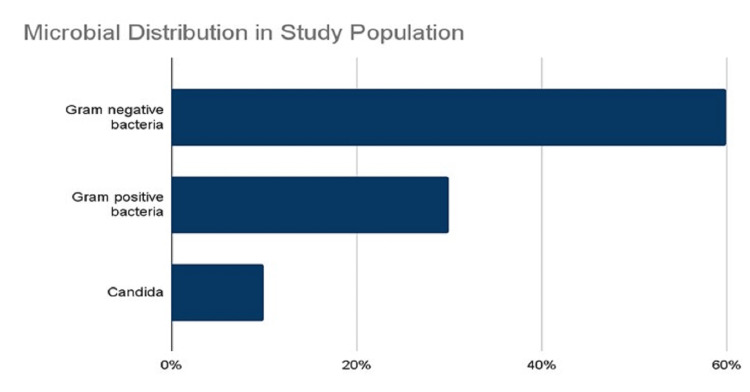
Broad classification of uropathogens isolated in the present study

A total of 158 patients were included in the study with 113 (71.5%) females and 45 (28.5%) males. A total of 95 (60%) cases had gram-negative microbes and 47 (30%) had gram-positive cocci, and 16 (10%) had candida infection (Figure 1).

Table 1 illustrates the uropathogen distribution isolated on culture. The most common pathogens included E.coli (gram-negative), methicillin resistant staphylococcus aureus (MRSA), and candida species.

**Table 1:** Microbe Isolated in Urine Culture

Name of Bacteria	%
Gram Negative Bacteria	
E-coli	54 (56.8%)
Klebsiella	17 (18.1%)
Serratia	19 (20.%)
Pseudomonas aeruginosa	5 (5.3%)
Total gram negative	95 (60%)
Gram Positive Cocci: Staphylococcus Aureus	47 (30%)
Candida	16 (10%)

As displayed in Table 2, in our current study the highly prevalent uropathogenic gram-positive bacteria showed the highest sensitivity to methicillin, rifampicin, vancomycin, and linezolid. Methicillin-resistant staph aureus was detected in 25% of isolates. All isolates of candida were sensitive to fluconazole. Gram-negative bacteria were highly resistant to ceftazidime, and cefepime.

**Table 2:** Pattern of Sensitivity and Resistance against bacteria specifically E.Coli and Staphylococcus Aureus (NT: not tested)

Antibiotic	Gram-negative Bacteria		Gram-positive Bacteria		Candida (Fungi)	
	Sensitivity (%)	Resistance (%)	Sensitivity (%)	Resistance (%)	Sensitivity (%)	Resistance (%)
Penicillin	NT	NT	30%	70%	NT	NT
Cefoxitin	NT	NT	41%	59%	NT	NT
Gentamicin	50%	50%	58%	42%	NT	NT
Fosfomycin	90%	10%	NT	NT	NT	NT
Amikacin	90%	10%	NT	NT	NT	NT
Nitrofurantoin	88%	12%	NT	NT	NT	NT
Meropenem	96%	4%	NT	NT	NT	NT
Imipenem	97%	3%	NT	NT	NT	NT
Sulbactam	60%	40%	NT	NT	NT	NT
Ceftriaxone	30%	70%	NT	NT	NT	NT
Ciprofloxacin	60%	40%	51%	49%	NT	NT
Cefixime	40%	60%	NT	NT	NT	NT
Fluconazole	NT	NT	NT	NT	100%	0%
Vancomycin	NT	NT	97%	3%	NT	NT
Linezolid	NT	NT	100%	0%	NT	NT
Trimethoprim / Sulfamethoxazole	NT	NT	40%	60%	NT	NT
Methicillin	NT	NT	75%	25%	NT	NT
Rifampicin	NT	NT	70%	30%	NT	NT
Piperacillin	50%	50%	NT	NT	NT	NT
Tazobactam	50%	50%	NT	NT	NT	NT
Ceftazidime	30%	70%	NT	NT	NT	NT
Cefepime	20%	80%	NT	NT	NT	NT
Clindamycin	NT	NT	20%	80%	NT	NT

## Discussion

Infections caused by multi-drug resistant microbes must be carefully monitored and treated by using already available broad-spectrum antibiotics as there are limited chances of the development of novel anti-infectives in the future [14].

In the present study, S. aureus was found to have the highest sensitivity against linezolid, and vancomycin and the lowest sensitivity to clindamycin and penicillins, similarly, the E.Coli showed the highest sensitivity to meropenem, imipenem, and fosfomycin while the lowest sensitivity to cefepime, ceftriaxone, and ceftazidime. 

Our study is in accordance with the literature available. In a retrospective study, electronic medical records of 4,284 (61.4% women) cUTI-related hospitalizations from 2008 to 2013 were analyzed [15]. The average patient age was 61.1 years and the median hospital stay was 11 days. One thousand and seventy-one urine and 148 blood specimens were cultured positive for different pathogens. Escherichia coli (48.2%), Klebsiella pneumoniae (9.5%), Pseudomonas aeruginosa (4.9%), and Proteus mirabilis (4.6%) were the most common gram-negative bacteria, while Enterococcus spp. (14.4%) was the most frequent gram-positive bacterium causing UTIs. High resistance rates (> 45%) to wide-spectrum penicillins, cephalosporins, aztreonam, and ciprofloxacin were seen in both E. coli and K. pneumoniae. Beta-lactamase inhibitor/beta-lactam antibiotic combination showed relatively lower rates of resistance. E. coli and K. pneumoniae showed the greatest sensitivity against Imipenem, meropenem, and amikacin [15].

Clinicians must take institutional-specific resistance tendencies into account while choosing empiric treatment. Additionally, host-specific factors such as prior anti-infective exposure, the severity of the signs and symptoms, history of allergies, and organ failure affect the choice of therapy [16].

Since patients with severe cUTIs may present with poor gastrointestinal tract function, vomiting, or diarrhea, which makes the drug therapy through the enteral route ineffective, empiric therapy should be delivered intravenously (IV). Therapy should be expedited as necessary once findings on culture and sensitivity are obtained [1,17]. Familiarization with the principles of antimicrobial use and management of associated urologic and medical comorbidities can help clinicians treat patients with cUTIs.

In a study conducted by Birru et al., the microbial resistance patterns were evaluated in patients with bloodstream infections. It was revealed that gram-positive isolates developed more diverse patterns of resistance and susceptibility. Resistance levels among gram-positive isolates ranged from 25 to 76.9 percent. Out of 13 isolates,10 (76.9%) and 8(61.5%) were resistant to penicillin and doxycycline respectively. The bacteria only exhibited lower or medium levels of resistance, i.e., 1/4 (25%); 4/13 (30.7%); 3/9 (33.3%); 4/9 (44.4%); and 6/13 (46.1%) against a variety of antibiotics, including vancomycin, erythromycin, gentamicin, tetracycline, ciprofloxacin, and chloramphenicol, respectively [18].

According to the findings of a study conducted in Addis Ababa, only 50% of the P. aeruginosa isolates were sensitive to piperacillin [19]. In contrast, meropenem was active against all of them and is also revealed by a study done in Nepal [20]. In line with our study, a research conducted in Pakistan, it was found that out of cumulative positive cultures, approximately 20% were gram-positive while almost four-fifth of the cultures yielded gram-negative bacteria. The frequently isolated pathogens were Escherichia coli (41.4%), Klebsiella, and pneumoniae (15.5%). Moreover, it was found that gram-negative bacteria were sensitive to Cefepime and all gram-positive microbes were sensitive to meropenem [11].

Despite the valuable contribution of the current study to the available literature, there are some limitations. For instance, the study's shorter duration, cross-sectional methodology, and smaller sample size all contribute to the difficulty of extrapolating the findings to a wider population. Therefore, we advise that future research should focus on a wider population and use information gathered from various centers.

Due to the indiscriminate use of antibiotics, the widely isolated uropathogens have a shifting resistance pattern, reducing the efficacy and safety of the treatment. In order to choose the best course of action for treating UTIs and preventing complications, antibiotic susceptibility patterns must be regularly and sporadically assessed.

## Conclusions

The development of bacterial resistance against multiple antibiotics is a global crisis that restricts the drug of choice for the treatment of cUTIs. In our study, we showed that overall, E. coli and S. aureus showed variable resistance to many antibiotics including methicillin, rifampicin, piperacillin, tazobactam, ceftazidime, cefepime, penicillin, cefoxitin, and gentamicin.
